# Immunogenicity and safety of COVID-19 vaccines among people living with HIV: A systematic review and meta-analysis

**DOI:** 10.1017/S095026882300153X

**Published:** 2023-09-14

**Authors:** Tianyu Zhao, Zongxing Yang, Yuxia Wu, Jin Yang

**Affiliations:** 1 Institute of Hepatology and Metabolic Diseases, Hangzhou Normal University, Hangzhou, China; 2 The Second Department of Infectious Disease, Xixi Hospital of Hangzhou, The Affiliated Hospital of Zhejiang Chinese Medical University, Hangzhou, China; 3 Department of Translational Medicine Center, Affiliated Hospital of Hangzhou Normal University, Hangzhou, China

**Keywords:** COVID-19, HIV, immunization (vaccination), safety, vaccines

## Abstract

Available data suggest that the immunogenicity of COVID-19 vaccines might decrease in the immunocompromised population, but data on vaccine immunogenicity and safety among people living with HIV (PLWH) are still lacking. The purpose of this meta-analysis is to compare the immunogenicity and safety of COVID-19 vaccines in PLWH with healthy controls. We comprehensively searched the following databases: PubMed, Cochrane Library, and EMBASE. The risk ratio (RR) of seroconversion after the first and second doses of a COVID-19 vaccine was separately pooled using random-effects meta-analysis. Seroconversion rate was lower among PLWH compared with healthy individuals after the first (RR = 0.77, 95% conﬁdent interval (CI) 0.64–0.92) and second doses (RR = 0.97, 95%CI 0.95–0.99). The risk of total adverse reactions among PLWH is similar to the risk in the healthy group, after the first (RR = 0.87, 95%CI 0.70–1.10) and second (RR = 0.83, 95%CI 0.65–1.07) doses. This study demonstrates that the immunogenicity and safety of SARS-CoV-2 vaccine in fully vaccinated HIV-infected patients were generally satisfactory. A second dose was related to seroconversion enhancement. Therefore, we considered that a booster dose may provide better seroprotection for PLWH. On the basis of a conventional two-dose regimen for COVID-19 vaccines, the booster dose is very necessary.

## Introduction

The COVID-19 pandemic caused severe global morbidity and mortality. By 6 April 2023, more than 760 million people had been infected with SARS-CoV-2 and more than 6.8 million deaths had occurred worldwide (https://covid19.who.int). Currently, omicron is the globally dominant variant, and the COVID-19 pandemic is mainly brought about by the emerging BA.2 and BA.5 sub-lineages [1]. Omicron has a remarkable capacity for immune evasion and has evolved numerous variants [2]. It is capable of infecting previously infected and vaccinated people [1].

The COVID-19 vaccines were developed on different platforms, and they play a major role in controlling the SARS-CoV-2 pandemic [3]. Authorised vaccines for COVID-19 to date include the mRNA vaccines (BNT162b2 and mRNA-1273), the adenoviral-vectored vaccines (ChAdOx1 nCoV-19 and Ad26.COV2.S), and the inactivated vaccines (BBIBP-CorV and CoronaVac) [4]. As of 6 April 2023, more than 13.3 billion SARS-CoV-2 vaccines have been administered worldwide, and the mRNA vaccines are the most commonly used. An inspiring example is that the COVID-19 vaccine has achieved great results in preventing infection and the development of severe disease in countries with high coverage rates [5]. The team of Netto conducted a cohort study to explore the safety and immunogenicity of CoronaVac among PLWH. The results suggested that the rates of seroconversion and neutralising antibody (Nab) positivity were high in HIV-positive people and no serious adverse reactions were reported [6].

Although numerous studies about the COVID-19 vaccination have been conducted, there are limited valid data among PLWH. Based on the World Health Organization report, HIV infection is a relevant risk factor for the severity of novel coronavirus infection and might be related to higher mortality [7]. HIV infection leads to a significant loss of CD4+ T cells by compromising the immune system. Pathogenicity mechanisms include impairing humoral and cellular responses and causing immune activation [8]. Ultimately, the immunogenicity of various vaccines was reduced. PLWH are highly susceptible to infections, particularly in those that are not on antiretroviral therapy (ART) and severe immunosuppression, putting them at risk of opportunistic infections [9, 10]. Therefore, vaccination is an important preventative measure for disease occurrence, and ensuring the efficacy of the vaccine in disease prevention is of crucial importance.

Research studies have indicated that the effects of current vaccines such as hepatitis B virus vaccines (HBV vaccines), influenza vaccines, and pneumococcal vaccines in the PLWH are different. In a systematic review of the immunogenicity of influenza vaccines among HIV-positive people, evidence suggests that influenza vaccines provide excellent seroconversion and seroprotection outcomes [11]. In the other study, a double dose of the HBV vaccines is significantly more efficacious than a standard dose of HBV vaccines in PLWH [12]. Miiro et al. performed a study indicating that the 7-valent conjugate pneumococcal vaccine showed good immunogenicity in HIV-infected adults [13]. These findings may help further research evaluating novel vaccination strategies, especially guiding PLWH for COVID-19 vaccination.

Kang et al. published a meta-analysis on the immunogenicity and safety of COVID-19 vaccines among HIV-infected patients [14]. The meta-analysis showed that the risk of achieving seroconversion was not significantly different between PLWH and healthy controls after the first and second doses of COVID-19 vaccines. However, our studies yielded inconsistent results, and the immunogenicity of COVID-19 vaccines is an attractive topic that is worthy of further exploration. We compared seroconversion between PLWH and healthy individuals in this meta-analysis, according to different COVID-19 vaccines. Our study will provide evidence-based references for PLWH regarding COVID-19 vaccines.

## Methods

This systematic review was carried out according to the Preferred Reporting Items for Systematic Reviews and Meta-Analysis guidelines [15]. This study has been registered on PROSPERO under the number PROSPERO CRD 42023410760.

### Literature search

We searched three databases: PubMed, EMBASE, and Cochrane Library (between 1 January 2020 and 19 March 2023) for relevant studies. The search terms used were as follows: (“COVID-19” or “Coronavirus” or “SARS-CoV-2”) and (“HIV infections” or “acquired immunodeficiency syndrome” or “HIV”) and (“Vaccination” or “Vaccines”). Detailed retrieval strategies are shown in Supplementary material S1. Supplementary material is available on the Cambridge Core website. One reviewer (T.Z.) performed the title and abstract screening, and the full text of the included studies was reviewed independently by two reviewers (Z.Y. and T.Z.), with potential discrepancies resolved by a third reviewer (J.Y.). No language or publication date restrictions were applied. To ensure data accuracy, non-peer-reviewed articles were excluded.

### Inclusion and exclusion criteria

Inclusion criteria were set as follows: (1) observational studies (cohort studies, case–control studies, and cross-sectional studies), RCTs, and non-randomised controlled trials; (2) studies with extractable data on seroconversion rates of Nab and incidence rates of adverse events; and (3) studies reporting PLWH receiving any COVID-19 vaccines who had never been infected with SARS-CoV-2.

Exclusion criteria were set as follows: (1) any other study design such as letters, comments, case reports, reviews, and animal experiments; (2) preprint articles; (3) full text was not available; (4) studies that did not report an HIV-negative control group; and (5) studies that did not provide sufficient data (seroconversion rates of Nab and incidence rates of adverse events).

### Data extraction

Eligible studies were independently evaluated by two researchers (T.Z. and Z.Y.). At the end of the data extraction phase, all key extracted data were reviewed and quality checked by the same two researchers.

The following data were extracted from each included study: (1) basic information about the studies, including date of publication, first author, region, and study design; (2) relevant information about COVID-19 vaccines, involving vaccine types, dosing schedule, and the interval between last dose and antibody testing; (3) immunogenicity outcome, including the number of HIV-infected participants with seroconversion of Nab (anti-RBD-IgG or anti-spike IgG); and (4) safety outcome: the number of adverse reactions among PLWH. We also collected data about healthy individuals in eligible studies, including the number of healthy groups, seroconversion rates, and adverse event incidence.

### Quality assessment

Two reviewers (T.Z. and Z.Y.) independently assessed the risk of bias in the included studies. We used the revised Cochrane risk-of-bias tool [16] for randomised controlled trials (RCTs) to assess the risk of bias. For non-randomised clinical trials, we used the Non-Randomized Studies of Interventions (ROBINS-I) tool [17]. For cohort studies and case–control studies, we used the Newcastle–Ottawa scale. For cross-sectional studies, we used the Agency for Healthcare Research and Quality (AHRQ).

### Statistical analysis

The meta-analysis was carried out using Review Manager Version 5.2 software and STATA version 17.0. We calculated the RR and 95% CI using a random-effects model to analyse primary outcomes of interest. An RR value <1 demonstrates that there is a reduced risk of seroconversion among PLWH who complete the vaccination, compared with healthy controls. We inspected heterogeneity among studies by I^2^ statistic, and I^2^ statistic ≥50% was considered to have significant heterogeneity. Meta-regression analysis and subgroup analysis were performed for potential sources of between-study heterogeneity. We used sensitivity analysis to assess the robustness of the primary outcome. Besides, publication bias was assessed by the funnel plot and Egger’s test.

## Results

### Study selection

**Figure 1.**
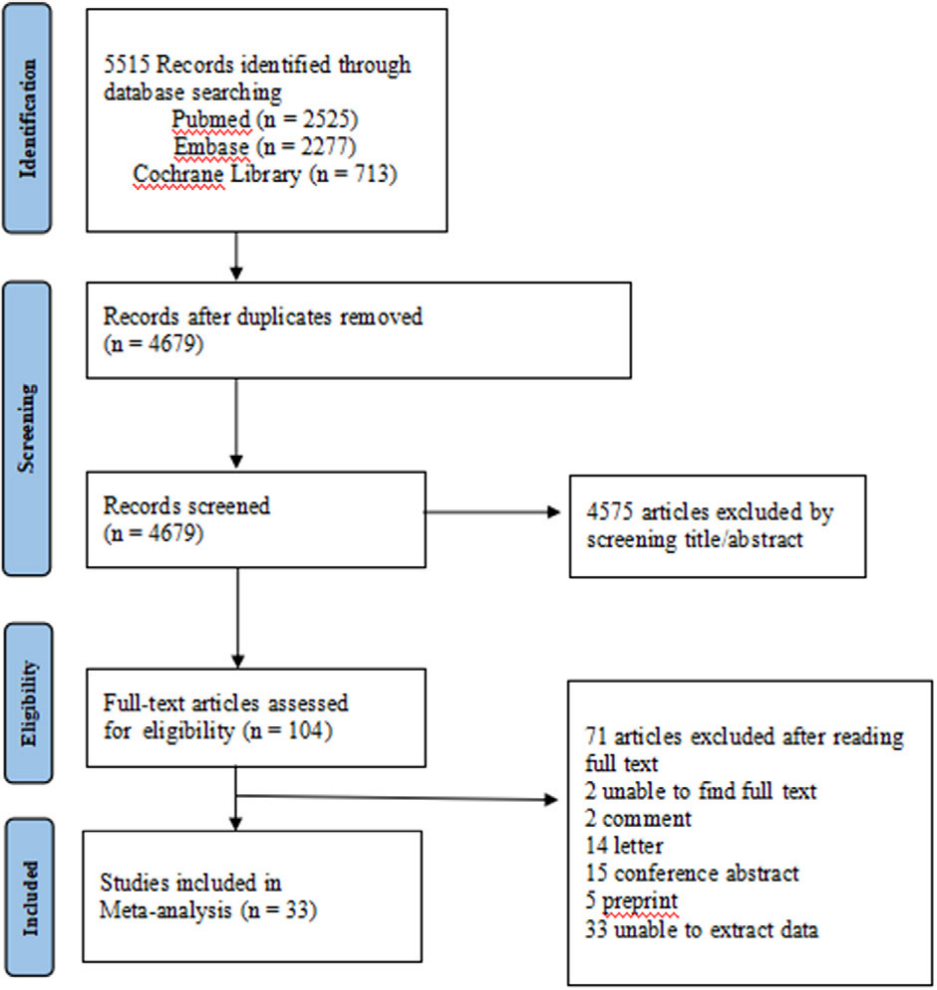
Flow chart of study selection.

The selection flow chart is displayed in Figure 1. A total of 5515 studies were identified through the database search, and 836 duplicates were deleted. About 4575 studies were deemed irrelevant after reviewing the titles and abstracts. Of the 104 studies, 71 were excluded based on the exclusion criterion. Therefore, 33 studies included in the meta-analysis met the inclusion criteria: 27 articles [18–44] for only immunogenicity and six articles for both immunogenicity and safety [6, 45–49].

### Characteristics of the included studies

Of the 33 included studies, 14 (42.4%) studies involved mRNA vaccines [BNT162b2 or mRNA-1273], 11 (33.3%) inactivated vaccines [BBIBP-CorV or CoronaVac], two (6.1%) adenovirus vector vaccines [AZD1222], one (3.0%) recombinant spike protein nanoparticle vaccine [MVC-COV1901], and five (15.2%) more than two vaccine types. Among the 33 studies, 23 (69.7%) were cohort studies, four (12.1%) were cross-sectional studies, four (12.1%) were non-randomised controlled trials, one (3.0%) was an RCT, and one (3.0%) was a case–control study. Fourteen (42.4%) studies were carried out in Asia, five (15.2%) in North America, 12 (36.4%) in Europe, one (3.0%) in South America, and one (3.0%) in Africa. The characteristics of the included studies are shown in Supplementary Tables S1–S3.

### Seroconversion rates among PLWH versus healthy controls

**Figure 2.**
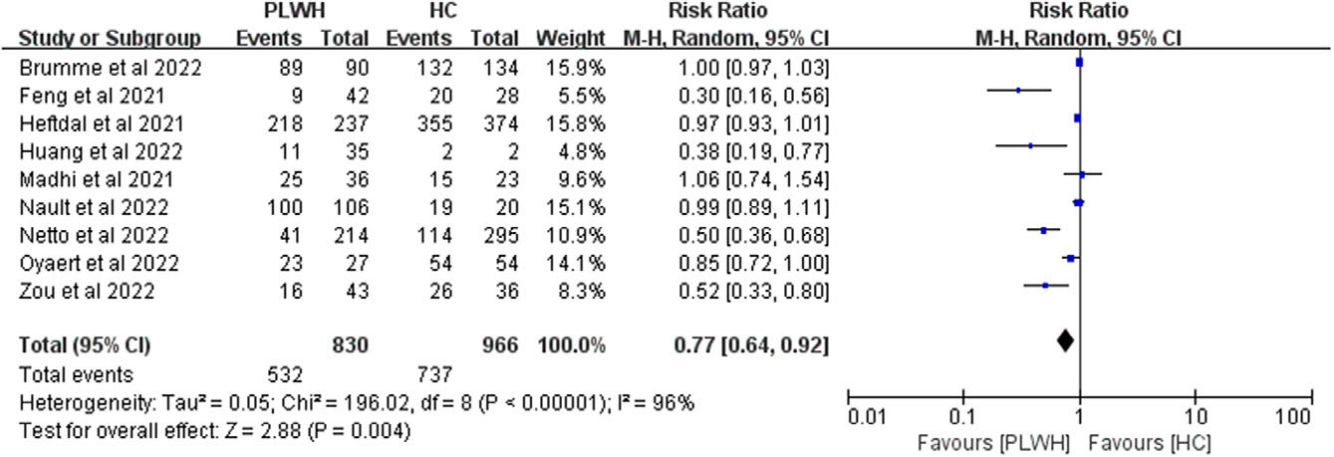
Risk ratios for seroconversion among PLWH compared with healthy controls after the first dose of the COVID-19 vaccine. Abbreviations: CI, confidence interval; HC, healthy controls; M-H, Mantel–Haenszel; PLWH, people living with HIV.

Nine studies reported seroconversion among PLWH (n = 830) compared with the HIV-negative group (n = 966) after the first dose. After the first vaccine dose, seroconversion rates were lower in the HIV-positive patients than in healthy individuals (RR 0.77, 95% CI 0.64–0.92, I^2^ = 96%, Figure 2).

**Figure 3.**
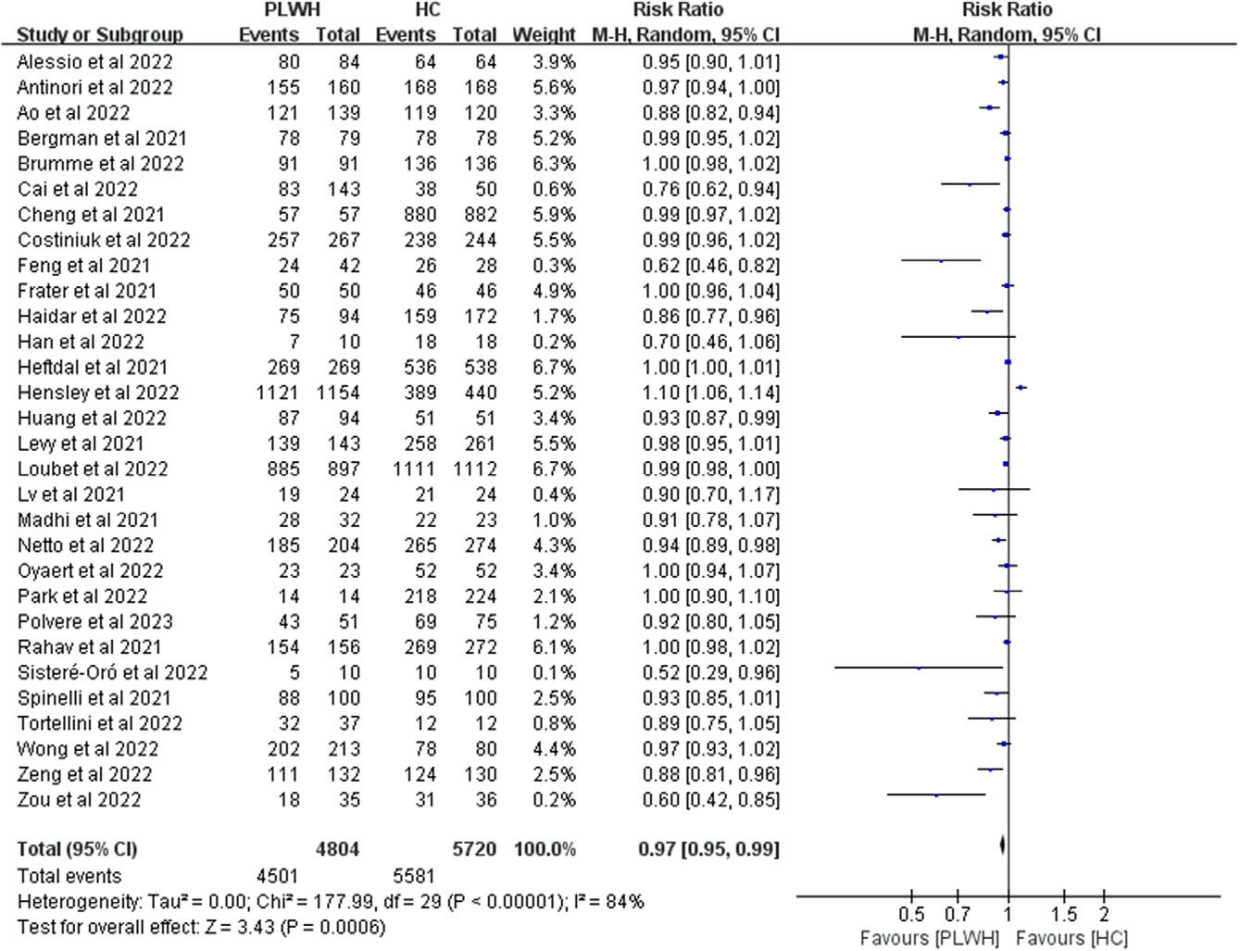
Risk ratios for seroconversion among PLWH compared with healthy controls after the second dose of the COVID-19 vaccine. Abbreviations: CI, confidence interval; HC, healthy controls; M-H, Mantel–Haenszel; PLWH, people living with HIV.

Thirty studies reported seroconversion among PLWH (n = 4804) compared with the HIV-negative group (n = 5720) after the second dose. After the second vaccine dose, seroconversion rates were lower in the HIV-infected patients than in healthy individuals (RR 0.97, 95% CI 0.95–0.99, I^2^ = 84%, Figure 3).

Five studies including 651 PLWH and 419 healthy individuals showed the results of immunogenicity after a booster dose. After a third dose, the seroconversion rates of PLWH were similar to those of healthy individuals (RR 0.97, 95% CI 0.90–1.04, I^2^ = 88%, Supplementary Figure S1).

### Subgroup analysis and heterogeneity test results

**Figure 4.**
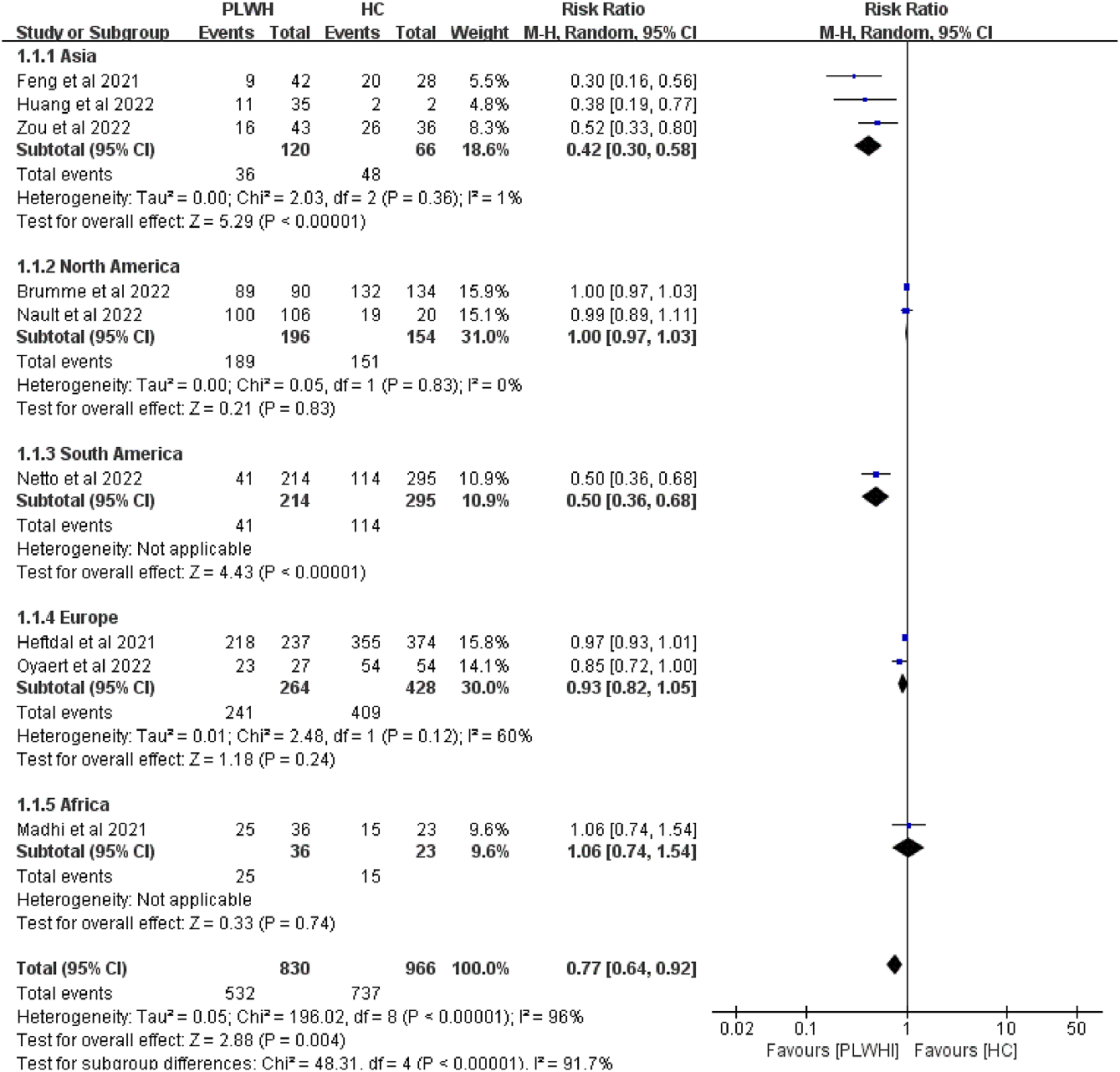
Subgroup analysis of different continents among PLWH compared with HC after the first dose of a COVID-19 vaccine. Abbreviations: CI, confidence interval; HC, healthy controls; M-H, Mantel–Haenszel; PLWH, people living with HIV.

The subgroup analysis was conducted for studies among vaccine types, study designs, and different regions. After the first dose, significant differences were observed in different regions (*P* < 0.05, Figure 4). Meta-regressions were performed to clarify the sources of heterogeneity among studies. In the analysis of seroconversion rates among PLWH compared with healthy individuals, regions (*P* < 0.05) might contribute to the heterogeneity.

**Figure 5.**
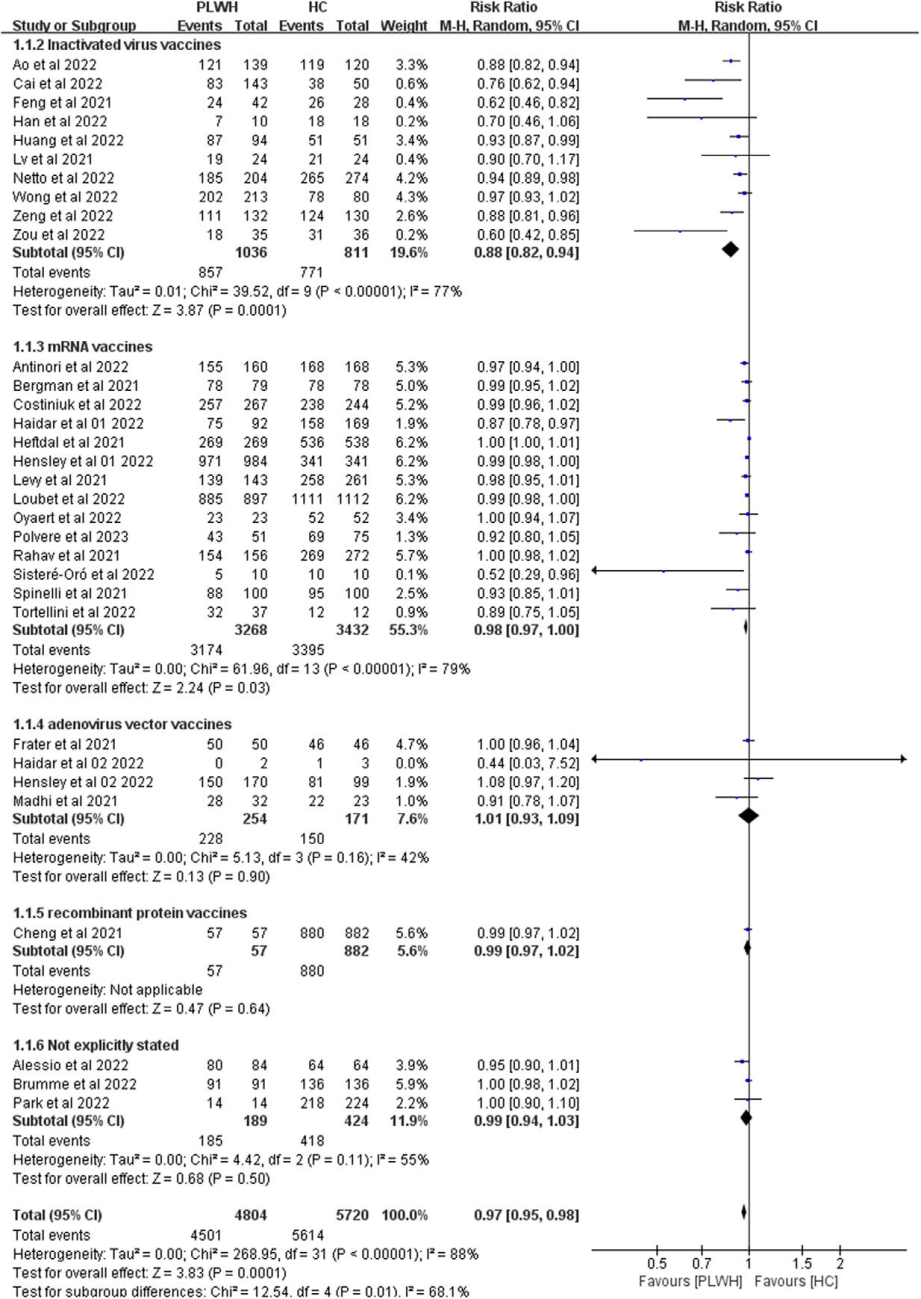
Subgroup analysis of vaccine type among PLWH compared with HC after the second dose of a COVID-19 vaccine. Abbreviations: M-H: Mantel–Haenszel; PLWH: people living with HIV; HC: healthy controls; CI: confidence interval.

After the second dose, significant subgroup differences were found in vaccine types (*P* = 0.01, I^2^ = 68.1%, Figure 5), and differences in RR among different continents (*P* = 0.03, I^2^ = 64.0%, Supplementary Figure S2) were also significant. We used meta-regression analysis, and the results suggest that vaccine types (*P* = 0.013) might contribute to the heterogeneity.

### Sensitivity analysis

To clarify the heterogeneity in seroconversion observed after the first and second doses, we performed a sensitivity analysis by deleting the literature one by one. After the first vaccine dose, we found that Heftdal’s study might be the source of heterogeneity (Supplementary Figure S3). After the second vaccine dose, by excluding the merged studies one by one, the effect sizes and values of the remaining studies did not change significantly, compared with the original studies. Sensitivity analysis suggests that the results are relatively stable (Supplementary Figure S4).

### Publication bias

**Figure 6.**
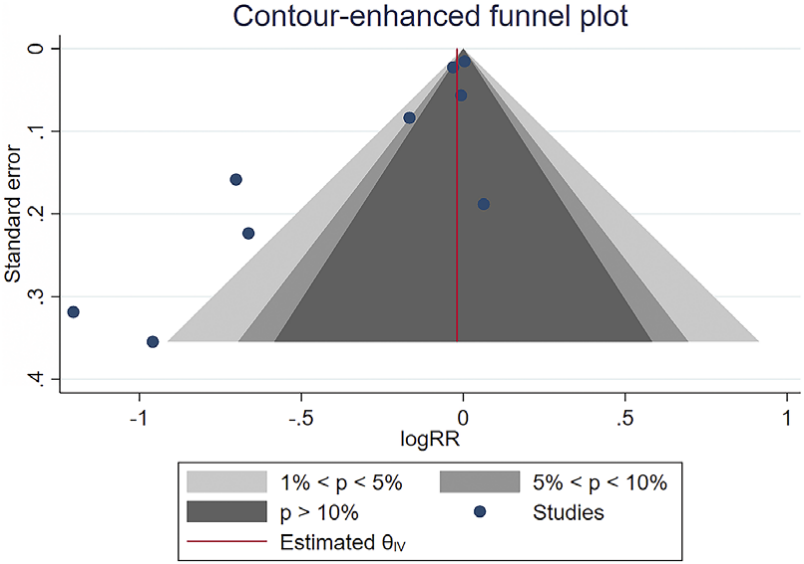
Funnel plot for studies of seroconversion among people living with HIV compared with healthy controls after the first dose of the COVID-19 vaccine.

**Figure 7.**
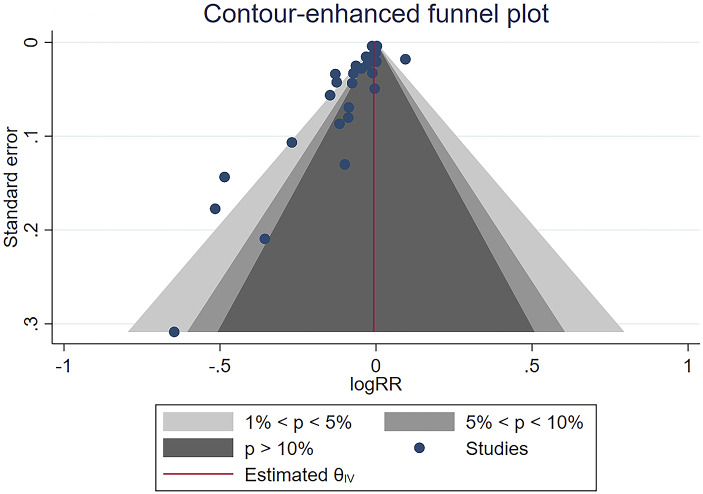
Funnel plot for studies of seroconversion among people living with HIV compared with healthy controls after the second dose of the COVID-19 vaccine.

Publication bias analysis was conducted using the funnel plot method for the change in the value of risk ratios for seroconversion after both doses. The funnel plots were all asymmetrically distributed, indicating the presence of publication bias (Figures 6 and 7). Publication bias was also examined with Egger’s test. A *P-*value less than 0.05 was considered to be a high probability of publication bias.

### Safety of COVID-19 vaccines in PLWH

Five articles assessed side effects after achieving the first dose, involving 490 PLWH and 2053 healthy controls. The relative risk of total adverse events (RR = 0.87, 95% CI 0.70–1.10) and systemic adverse events (RR = 0.92, 95% CI 0.80–1.05) among HIV-infected patients did not differ from healthy individuals (Supplementary Figures S5 and S7). Compared to healthy individuals, the relative risk of local adverse reactions (RR = 0.73, 95% CI 0.57–0.95) among the PLWH was lower (Supplementary Figure S9).

Six articles assessed side effects after achieving the second dose, involving 646 PLWH and 2095 healthy controls. The relative risk of total adverse events (RR = 0.83, 95% CI 0.65–1.07) and local adverse events (RR = 0.65, 95% CI 0.38–1.12) among PLWH did not differ from the healthy group (Supplementary Figures S6 and S10). The relative risk of systemic adverse events in the PLWH was lower (RR = 0.80, 95% CI 0.69–0.93) than in the HIV-negative group (Supplementary Figure S8).

## Discussion

A large number of clinical trials proved that vaccinations led to a decline in the risk of hospitalisations and COVID-19-associated infection. This systematic review aims to comprehensively assess the immunogenicity of COVID-19 vaccines among HIV-infected patients compared with healthy volunteers. In this meta-analysis of 33 studies, the pooled seroconversion rate among PLWH was lower than that of healthy individuals after the first and second vaccine doses. After the second dose, the humoral immune response (93.7%) among PLWH was slightly inferior to the humoral immune response (97.6%) among the healthy population. Compared with the first dose, seroconversion efficiency was higher after the second dose. At present, a definitive serological threshold for establishing protection through COVID-19 vaccination remains undefined. The most representative alternative indicator for assessing vaccine immunogenicity includes seroconversion rates and geometric mean titres. These alternative indicators generally involve many parameters related to SARS-CoV-2 spike protein, anti-RBD antibodies, neutralising IgG, or total antibodies. The use of antibodies to predict protection against COVID-19 has focused on the ability of the vaccine antibodies to bind to the virus, which partially reflects vaccine immunogenicity, but T-cell responses were not assessed [50]. Anti-RBD antibodies constitute a major part of neutralising antibody response [51]. However, the detection method employed in numerous studies exhibited variability, thereby rendering the determination of the optimal cut-off value inconclusive. Therefore, further studies and rational attempts are needed.

However, currently, systematic reviews on the same topic are scarce. Lee et al. [52] published a meta-analysis in immunocompromised patients to explore the efficacy of COVID-19 vaccines. There were no significant differences in seroconversion among PLWH compared with immunocompetent patients after the second dose (RR = 1.00, 95% CI 0.98–1.01). The findings of our study are inconsistent with the results of the study by Lee et al. We speculated that a few literature studies and the small sample size in the study by Lee et al. may have led to such a difference. A review demonstrated that the fourth dose was remarkable in elevating antibody titres among the immunocompromised population [53]. However, there are few published data on a fourth COVID-19 vaccination dose in PLWH [53]. Systematic reviews about the immunogenicity of booster dose among PLWH were still not published.

We performed subgroup analysis according to vaccine types to explore sources of heterogeneity. In the group vaccinated with the inactivated vaccine, the results demonstrated that the risk ratio for seroconversion among PLWH compared with healthy individuals (RR = 0.88, 95% CI 0.82–0.94) was the lowest after a second dose of the COVID-19 vaccine. To explore the possible source of between-study heterogeneity in different vaccine types, the meta-regression results suggest that vaccine types (*P* = 0.013) might contribute to the heterogeneity. Zheng et al. [54] provided synthesised evidence, which showed that the effectiveness of Moderna, Pfizer-BioNTech, and CoronaVac was 98.1%, 91.2%, and 65.7%, respectively. The inactivated vaccines had the lowest immunogenicity among a wide variety of types of COVID-19 vaccines. Our findings are consistent with the study by Zheng et al.

We analysed the PLWH population of the included studies and found that lower seroconversion rates were associated with a lower CD4 cell count. Vergori et al. [43] conducted a cohort study and found that NAb response that was defined as titres >1:10 was elicited in 86.3% of poor CD4 recovery (PCDR), 97.9% of intermediate CD4 recovery (ICDR), and 98.7% of high CD4 recovery (HCDR). Netto et al. [6] performed a prospective cohort study including 215 PLWH, and the results showed that PLWH with CD4+ T-cell counts of less than 500 cells/mm^3^ had lower seroconversion rates than those with CD4+ T-cell counts of at least 500 cells/mm^3^. Antinori et al. [25] initiated a nationwide prospective cohort study including 160 PLWH, and the result was that PLWH with CD4+ T-cell counts <200 cells/mm^3^ had a lower anti-RBD response, compared with PLWH with CD4+ T-cell counts >200 cells/mm^3^. Besides, we found that higher seroconversion rates were related to a lower viral load among PLWH. Highly active antiretroviral therapy (HAART) can suppress viral load, leading to immunologic recovery [55]. Therefore, suppressing viral load to increase CD4+ T-cell counts might improve vaccine-induced immunogenicity in PLWH. These findings indicated that strategies should be developed to improve vaccine-induced immunogenicity among PLWH, especially in the population with lower CD4+ T-cell counts and a higher HIV viral load.

In some Asian countries and regions, inactivated vaccines are customary and considered safe. The rate of mRNA vaccine vaccinations remains highest in the regions of Europe and America, and the adenovirus vector vaccine also has a high vaccination rate in these regions. In the subgroup analysis by regions, the pooled risk ratio for seroconversion among PLWH compared with healthy individuals after the second dose of the COVID-19 vaccine was lowest in Asia and highest in America and Europe. Our results suggested that the difference in seroresponse in different regions may be related to the regional distribution of the vaccine.

In our study, the safety of COVID-19 vaccines was not found to differ between HIV-infected patients and healthy controls. The risk of local adverse events (RR = 0.73, 95% CI 0.57–0.95) among PLWH was lower than the healthy controls after the first dose. The risk of systemic adverse reactions (RR = 0.80, 95% CI 0.69–0.93) in HIV-infected patients was also lower than in healthy individuals after the second dose. The source of the discrepancy may be caused by chance due to the relatively small number of studies. To clarify the difference in total adverse reactions after vaccination for different vaccine types, a meta-analysis that included 19 clinical trials showed that the pooled RRs of total adverse reactions for mRNA, inactivated, and vector vaccines were 2.01 (95% CI: 1.82–2.23), 1.46 (95% CI: 1.19–1.78), and 1.65 (95% CI: 1.31–2.32), respectively. The risk ratio of any adverse events was highest for mRNA vaccines and least for inactivated vaccines [56]. However, compared with other vaccine types, mRNA vaccines have the best efficacy so far, but the mechanism for developing adverse events remains unclear [57]. In summary, our results show that the COVID‐19 vaccines have good safety, the benefits of vaccination still outweigh the risks, and vaccination is recommended for all PLWH.

Furthermore, the majority of vaccine manufacturers and experts are paying attention to second-generation vaccines, such as bivalent vaccines and nasal vaccines. A bivalent vaccine, a traditional approach, provides broad coverage against two antigenically variable pathogens. Bivalent vaccine formulations were approved in Fall 2022 [58]. An animal study showed that both bivalent vaccines induced neutralising activity against BA.5 in BA.5-infected mice [59]. Midterm outcomes in a recent study showed that the bivalent vaccines performed better in neutralising capacity against omicron than mRNA vaccines broadly available [60]. Bivalent vaccines are a crucial strategy to mitigate the consequences of the spread of circulating variants and improve the immune protection of humans [59]. Currently, data are lacking on the efficacy of bivalent vaccines, and relevant clinical trials are ongoing [59].

As far as we know, this is the most comprehensive study to evaluate the COVID-19 vaccine’s immunogenicity and safety among PLWH. Sufficient studies published were included (from 1 January 2020 to 19 March 2023), and study subjects were PLWH receiving the first and second vaccine doses. Our findings could help alleviate vaccine hesitancy in PLWH and provide evidence-based decision-making.

This systematic review has some limitations. Firstly, the majority of studies are cohort studies, but four are non-randomised controlled trials and one is an RCT. Secondly, many factors can contribute to the heterogeneity among studies, such as sample size, vaccine type, study location, and the basic characteristics of the population. Thirdly, the seroconversion rate is an indicator only to predict the risk of severity of SARS-CoV-2 infection and evaluate the seroprotection effect. The SARS-CoV-2 infection rate is a significant indicator to assess clinical efficacy endpoints, but relevant studies are still lacking. Fourthly, some clinical studies suggested that additional doses have a great effect on improving antibody responses in PLWH, but further studies are needed to verify the results. Finally, seroconversion after vaccination differed considerably among a wide variety of vaccine types. Therefore, the vaccine type might have an effect on the results. Considering the included studies in this meta-analysis mainly used mRNA vaccines, the differential analysis was also limited.

## Conclusion

In conclusion, this meta-analysis demonstrates that the immunogenicity and safety of the SARS-CoV-2 vaccine among PLWH were satisfactory. The second dose was correlated with seroconversion improvement consistently; nevertheless, seroconversion rates were still lower in the PLWH group than in the healthy group. Additional strategies for improving vaccine efficacy in PLWH are needed. For example, a booster vaccine dose to the conventional two-dose regimen for mRNA vaccines would enhance seroprotection for these patients. Besides, promoting COVID-19 vaccination is urgent, and it is necessary to design more effective interventions to tackle vaccine hesitancy.

## Supporting information

Zhao et al. supplementary material Zhao et al. supplementary material

## Data Availability

The data that support the findings of this study are available from the corresponding author upon reasonable request.

## Supplementary material

For supplementary material accompanying this paper visit http://doi.org/10.1017/S095026882300153X. click here to view supplementary material
