# Supplementary material for: Immunogenicity and safety of COVID-19 vaccines among people living with HIV: A systematic review and meta-analysis
Source: Epidemiol Infect. 2023 Sep 14;151:e176. doi: 10.1017/S095026882300153X (PMC10600909; doi:10.1017/S095026882300153X)
Supplement: Zhao et al. supplementary material [file S095026882300153Xsup001.doc]

**Journal:** Epidemiology and Infection

**Title:** Immunogenicity and safety of COVID-19 vaccines among people living with HIV: A systematic review and meta-analysis

**Author names:** Tianyu Zhao, Zongxing Yang, Yuxia Wu, Jin Yang

**Supplementary Materials:**

Supplementary Text S1: Search strategy.

Supplementary figure S1. Risk ratios for seroconversion among PLWH compared with HC after a third dose of COVID-19 vaccine.

Supplementary figure S2. Subgroup analysis of different continents among PLWH patients compared with HC after second dose of a COVID-19 vaccine.

Supplementary figure S3. Sensitivity analysis of risk ratio of seroconversion by deleting the literature one by one (after first dose of a COVID-19 vaccine).

Supplementary figure S4. Sensitivity analysis of risk ratio of seroconversion by deleting the literature one by one (after second dose of a COVID-19 vaccine).

Supplementary figure S5. Risk ratios for Total adverse events among PLWH compared with HC after a first dose of COVID-19 vaccine.

Supplementary figure S6. Risk ratios for Total adverse events among PLWH compared with HC after a second dose of COVID-19 vaccine.

Supplementary figure S7. Risk ratios for Systemic adverse events among PLWH compared with HC after a first dose of COVID-19 vaccine.

Supplementary figure S8. Risk ratios for Systemic adverse events among PLWH compared with HC after a second dose of COVID-19 vaccine.

Supplementary figure S9. Risk ratios for Local adverse events among PLWH compared with HC after a first dose of COVID-19 vaccine.

Supplementary figure S10. Risk ratios for Local adverse events among PLWH compared with HC after a second dose of COVID-19 vaccine.

Supplementary Table S1: Characteristics and basic information of the studies included in the systematic review and meta-analysis for vaccine immunogenicity.

Supplementary Table S2: Baseline Characteristics of HIV-negative individuals and PLWH in the included studies

Supplementary Table S3: Characteristics and basic information of the studies included in the systematic review and meta-analysis for vaccine safety.

Supplementary Table S4: Risk of bias of all included randomized controlled trials (RCTs) using the RoB 2 scale.

Supplementary Table S5: Risk of bias of all included non-randomized clinical trials using the ROBINS-I scale.

Supplementary Table S6: Risk of bias of all included cohort studies using the Newcastle-Ottawa quality assessment scale.

Abbreviations: PLWH: people living with HIV; HC: healthy controls

**Supplementary Text S1: Search Strategy**

**Pubmed:**

**Search Terms:**

(“Coronavirus Infections”[MeSH] OR “Coronavirus”[MeSH] OR “SARS-CoV-2”[MeSH] OR “COVID-19”[MeSH]) OR (“2019 nCoV”[All Fields] OR 2019nCoV[All Fields] OR coronavir*[All Fields] OR coronovir*[All Fields] OR COVID[All Fields] OR COVID19[All Fields] OR HCoV*[All Fields] OR “nCov 2019”[All Fields] OR “SARS CoV2”[All Fields] OR “SARS CoV 2”[All Fields] OR SARSCoV2[All Fields] OR “SARSCoV 2”[All Fields] OR “severe acute respiratory syndrome coronavirus 2”[All Fields])

AND

(“HIV”[MeSH] OR “HIV infections”[MeSH] OR “anti-HIV agents”[MeSH] OR “HIV protease inhibitors”[MeSH] OR “reverse transcriptase inhibitors”[MeSH]) OR (HIV*[All Fields] OR human immun*[All Fields] OR “acquired immunodeficiency syndrome”[All Fields] OR “acquired immune deficiency syndrome”[All Fields] OR HIV infect*[All Fields] OR AIDS[All Fields])

AND

(“Vaccines”[MeSH] OR “Vaccination”[MeSH]) OR (Vaccin*[All Fields])

Search limits: 1 January 2020 to 19 March 2023

**Embase**

**Search Terms:**

('coronavirus Infection'/exp OR 'coronavirinae'/exp OR 'coronavirus disease 2019'/exp OR 'severe acute respiratory syndrome coronavirus 2'/exp OR ('coronavirinae' OR 'coronavirus Infection' OR 'coronavirus disease 2019' OR 'severe acute respiratory syndrome coronavirus 2' OR 'sars-cov*' OR 'coronavir*' OR 'covid*'))

AND

('human immunodeficiency virus'/exp OR 'human immunodeficiency virus infection'/exp OR 'anti human immunodeficiency virus agent'/exp OR 'human immunodeficiency virus proteinase inhibitor'/exp OR 'acquired immune deficiency syndrome'/exp OR ('human immunodeficiency virus' OR 'human immunodeficiency virus infection' OR 'HIV*' OR 'human immun*' OR 'acquired immune deficiency syndrome' OR 'AIDS'))

AND

('vaccination'/exp OR 'vaccine'/exp OR ('vaccin*'))

Search limits: 1 January 2020 to 19 March 2023

**Cochrane Library**

**Search Terms:**

(“Coronavirus Infections”[MeSH] OR “Coronavirus”[MeSH] OR “SARS-CoV-2”[MeSH] OR “COVID-19”[MeSH] OR (coronavir* OR covid-19 OR sars-cov* OR severe acute respiratory syndrome coronavirus 2))

AND

(“HIV”[MeSH] OR “HIV infections”[MeSH] OR “anti-HIV agents”[MeSH] OR (HIV* OR human immun* OR acquired immune deficiency syndrome OR AIDS))

AND

(vaccin*)

Search limits: 1 January 2020 to 19 March 2023

**Supplementary figure S1.** Risk ratios for seroconversion among PLWH compared with HC after a third dose of COVID-19 vaccine.


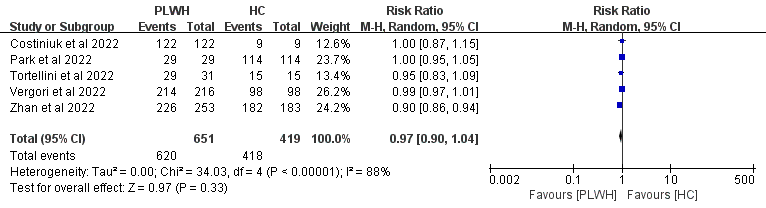
Abbreviations: M-H: Mantel-Haenszel; PLWH: people living with HIV; HC: healthy controls; CI: confidence interval

**Supplementary figure S2.** Subgroup analysis of different continents among PLWH compared with HC after second dose of a COVID-19 vaccine.


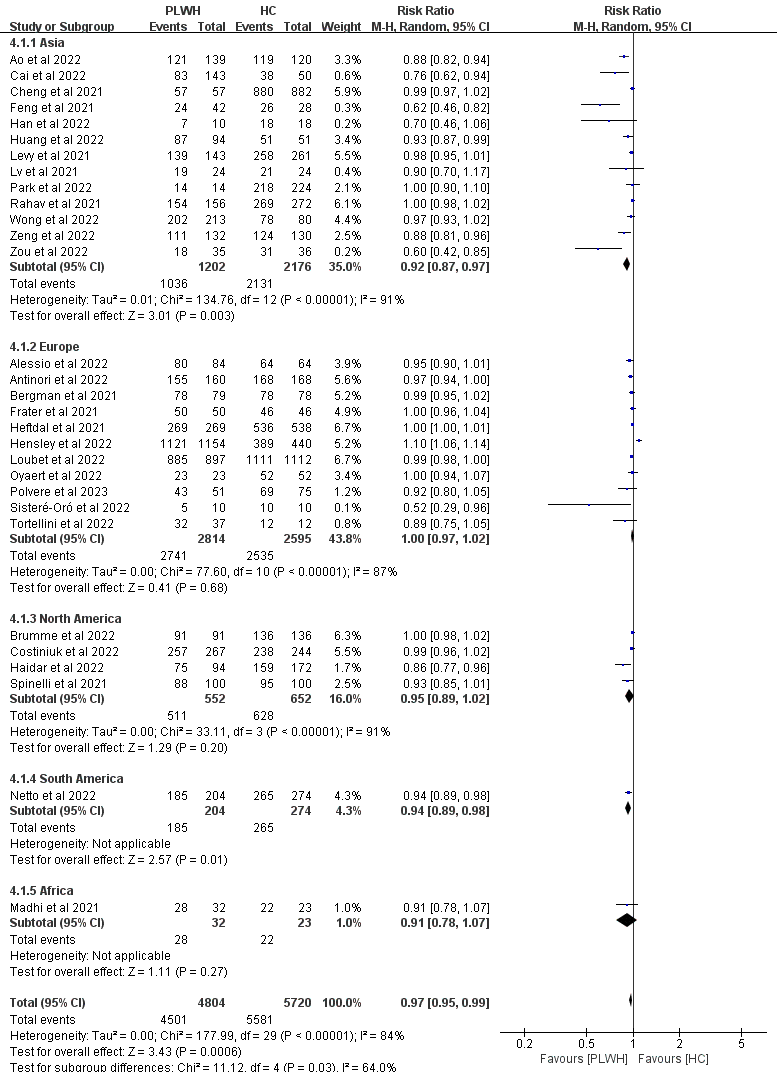
Abbreviations: M-H: Mantel-Haenszel; PLWH: people living with HIV; HC: healthy controls; CI: confidence interval

**Supplementary figure S3.** Sensitivity analysis of risk ratio of seroconversion by deleting the literature one by one (after first dose of a COVID-19 vaccine).


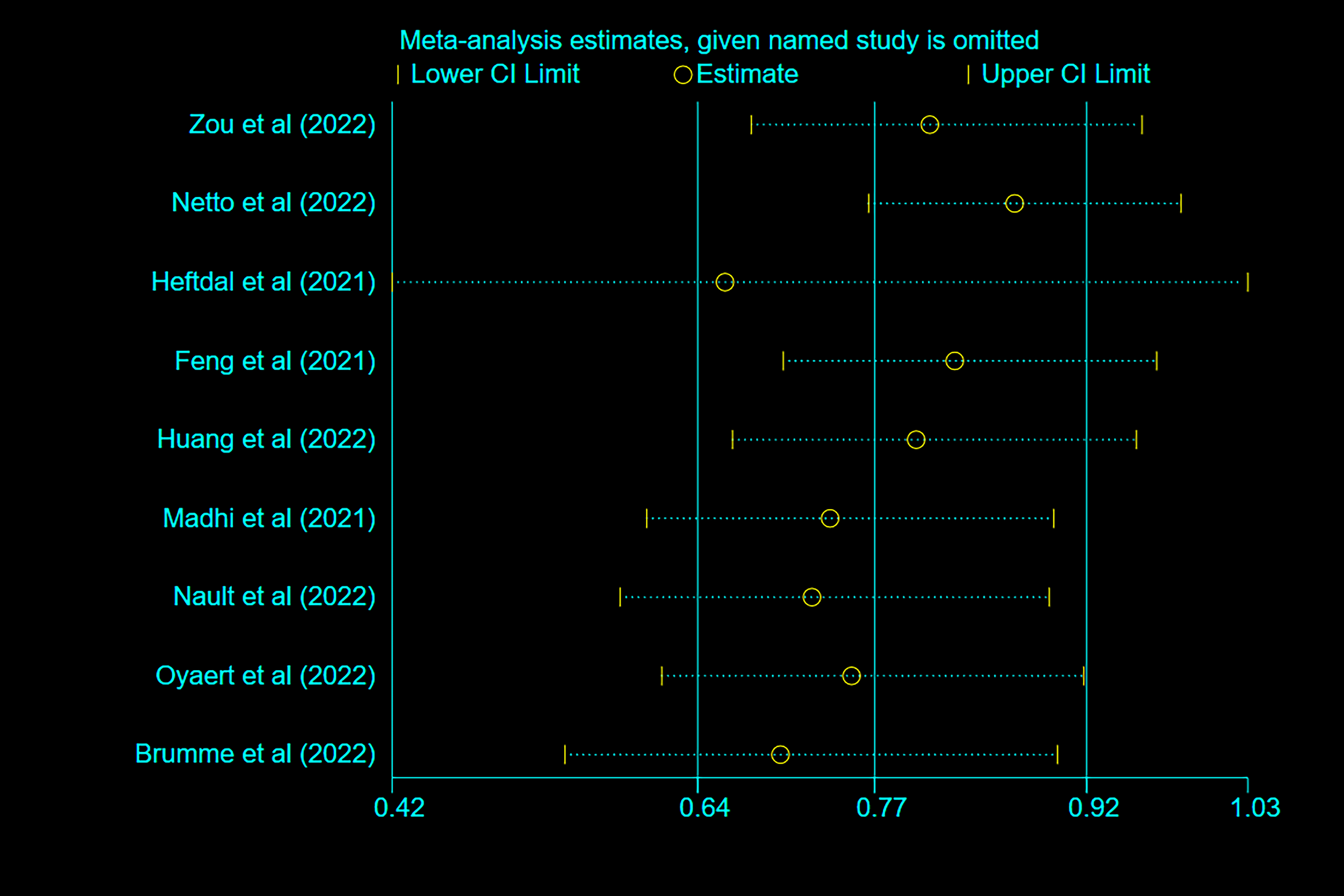
**Supplementary figure S4.** Sensitivity analysis of risk ratio of seroconversion by deleting the literature one by one (after second dose of a COVID-19 vaccine).


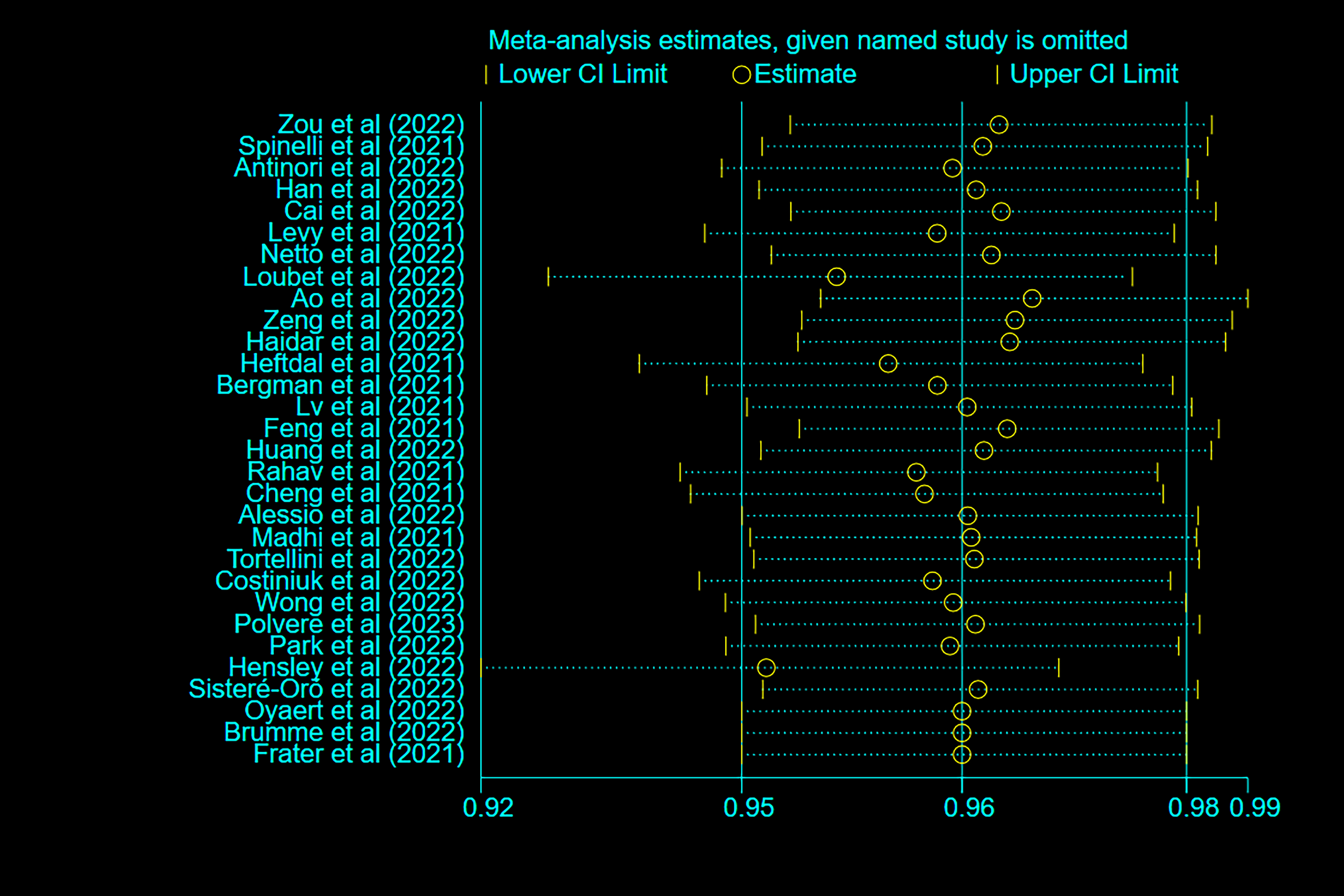


**Supplementary figure S5.** Risk ratios for Total adverse events among PLWH compared with HC after a first dose of COVID-19 vaccine.


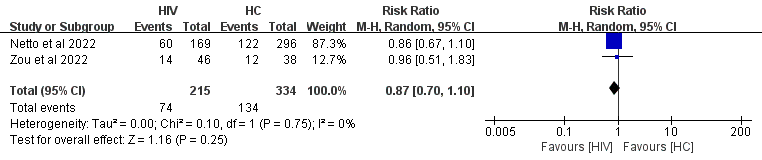
Abbreviations: M-H: Mantel-Haenszel; HC: healthy controls; PLWH: people living with HIV; CI: confidence interval

**Supplementary figure S6.** Risk ratios for Total adverse events among PLWH compared with HC after a second dose of COVID-19 vaccine.


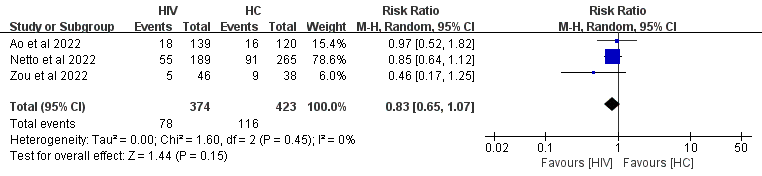
Abbreviations: M-H: Mantel-Haenszel; HC: healthy controls; PLWH: people living with HIV; CI: confidence interval

**Supplementary figure S7.** Risk ratios for Systemic adverse events among PLWH compared with HC after a first dose of COVID-19 vaccine.


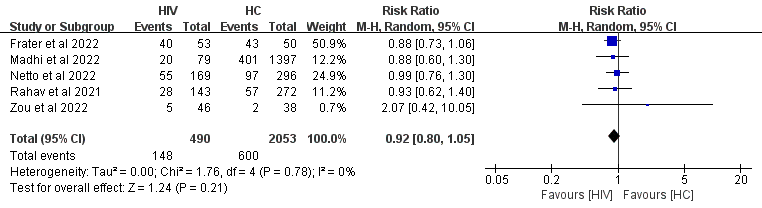
Abbreviations: M-H: Mantel-Haenszel; HC: healthy controls; PLWH: people living with HIV; CI: confidence interval

**Supplementary figure S8.** Risk ratios for Systemic adverse events among PLWH compared with HC after a second dose of COVID-19 vaccine.


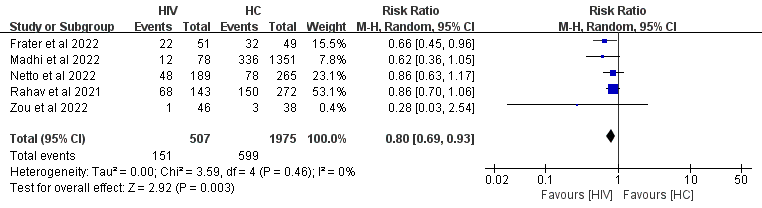
Abbreviations: M-H: Mantel-Haenszel; HC: healthy controls; PLWH: people living with HIV; CI: confidence interval

**Supplementary figure S9.** Risk ratios for Local adverse events among PLWH compared with HC after a first dose of COVID-19 vaccine.


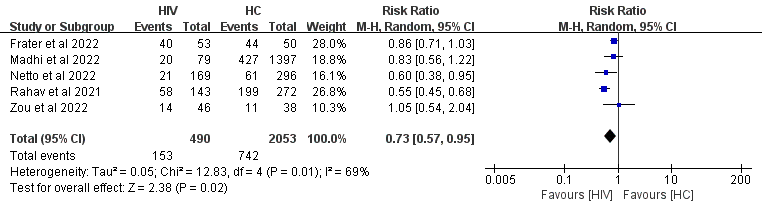
Abbreviations: M-H: Mantel-Haenszel; HC: healthy controls; PLWH: people living with HIV; CI: confidence interval

**Supplementary figure S10.** Risk ratios for Local adverse events among PLWH compared with HC after a second dose of COVID-19 vaccine.


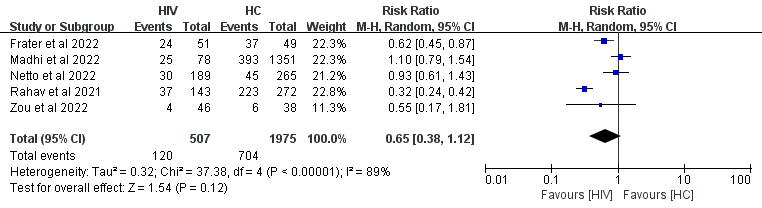
Abbreviations: M-H: Mantel-Haenszel; HC: healthy controls; PLWH: people living with HIV; CI: confidence interval

**Supplementary Table S1:** Characteristics and basic information of the studies included in the systematic review and meta-analysis for COVID-19 vaccine immunogenicity.

| Author | Publication  year | Study design | Region | Vaccine type | Vaccine dose | Time*  interval | PLWH with  seroconversion | PLWH | HC with  seroconversion | HC |
| --- | --- | --- | --- | --- | --- | --- | --- | --- | --- | --- |
| Zou et al | 2022 | cohort study | Asia | Sinopharm or WIBP-CorV | 1 | after 42 days | 16 | 43 | 26 | 36 |
| Zou et al | 2022 | cohort study | Asia | Sinopharm or WIBP-CorV | 2 | after 42 days | 18 | 35 | 31 | 36 |
| Spinelli et al | 2021 | case-control study | North America | BNT162b2 or mRNA1273 | 2 | after 35 days | 88 | 100 | 95 | 100 |
| Antinori et al | 2022 | cohort study | Europe | BNT162b2 or mRNA1273 | 2 | after 1 month | 155 | 160 | 168 | 168 |
| Han et al | 2022 | cohort study | Asia | Sinopharm or Sinovac | 2 | after 4 weeks | 7 | 10 | 18 | 18 |
| Cai et al | 2022 | cross-sectional study | Asia | Sinopharm or Sinovac | 2 | ≥ 5 days | 83 | 143 | 38 | 50 |
| Levy et al | 2021 | cohort study | Asia | BNT162b2 | 2 | after 18 days | 139 | 143 | 258 | 261 |
| Netto et al | 2022 | cohort study | South America | CoronaVac | 2 | after 6 weeks | 185 | 204 | 265 | 274 |
| Netto et al | 2022 | cohort study | South America | CoronaVac | 1 | after 4 weeks | 41 | 214 | 114 | 295 |
| Loubet et al | 2022 | cohort study | Europe | BNT162b2 or mRNA1273 | 2 | after 1 month | 885 | 897 | 1111 | 1112 |
| Ao et al | 2022 | cohort study | Asia | BBIBP-CorV or CoronaVac | 2 | ≥ 21 days | 121 | 139 | 119 | 120 |
| Zeng et al | 2022 | cohort study | Asia | BBIBP-Corv or CoronaVac | 2 | after 28 days | 111 | 132 | 124 | 130 |
| Haidar et al | 2022 | cohort study | North America | mRNA vaccine | 2 | ≥ 14 days | 75 | 92 | 158 | 169 |
| Haidar et al | 2022 | cohort study | North America | Viral vector vaccine | 2 | ≥ 14 days | 0 | 2 | 1 | 3 |
| Heftdal et al | 2021 | cohort study | Europe | BNT162b2 | 1 | after 14 days | 218 | 237 | 355 | 374 |
| Heftdal et al | 2021 | cohort study | Europe | BNT162b2 | 2 | after 7 days | 269 | 269 | 536 | 538 |
| Bergman et al | 2021 | non-randomized controlled trial | Europe | BNT162b2 | 2 | after 14 days | 78 | 79 | 78 | 78 |
| Lv et al | 2021 | non-randomized controlled trial | Asia | CoronaVac or BBIBP-CorV | 2 | after 40 days | 19 | 24 | 21 | 24 |
| Feng et al | 2021 | non-randomized controlled trial | Asia | BBIBP-CorV | 1 | after 4 weeks | 9 | 42 | 20 | 28 |
| Feng et al | 2021 | non-randomized controlled trial | Asia | BBIBP-CorV | 2 | after 4 weeks | 24 | 42 | 26 | 28 |
| Huang et al | 2022 | cross-sectional study | Asia | CoronaVac or Sinopharm | 1 | not stated | 11 | 35 | 2 | 2 |
| Huang et al | 2022 | cross-sectional study | Asia | CoronaVac or Sinopharm | 2 | at least several days | 87 | 94 | 51 | 51 |
| Rahav et al | 2021 | cohort study | Asia | BNT162b2 | 2 | after 2-4 weeks | 154 | 156 | 269 | 272 |
| Cheng et al | 2021 | cohort study | Asia | MVC-COV1901 | 2 | after 28 days | 57 | 57 | 880 | 882 |
| Alessio et al | 2022 | cross-sectional study | Europe | BNT162b2 or mRNA1273 or ChAdOx1 | 2 | after 1 month | 80 | 84 | 64 | 64 |
| Madhi et al | 2021 | RCT | Africa | AZD1222 | 1 | after 28 days | 25 | 36 | 15 | 23 |
| Madhi et al | 2021 | RCT | Africa | AZD1222 | 2 | after 14 days | 28 | 32 | 22 | 23 |
| Tortellini et al | 2022 | cohort study | Europe | BNT162b2 | 2 |  | 32 | 37 | 12 | 12 |
| Tortellini et al | 2022 | cohort study | Europe | BNT162b2 | 3 | after 2 months | 29 | 31 | 15 | 15 |
| Costiniuk et al | 2022 | cohort study | North America | BNT162b2 or mRNA1273 | 2 | after 3 months | 257 | 267 | 238 | 244 |
| Costiniuk et al | 2022 | cohort study | North America | BNT162b2 or mRNA1273 | 3 | after 4 weeks | 122 | 122 | 9 | 9 |
| Wong et al | 2022 | cohort study | Asia | CoronaVac | 2 | after 14-42 days | 202 | 213 | 78 | 80 |
| Vergori et al | 2022 | cohort study | Europe | BNT162b2 or mRNA1273 | 3 | after 15 days | 214 | 216 | 98 | 98 |
| Zhan et al | 2022 | cross-sectional study | Asia | inactivated vaccine | 3 | ≥ 14 days | 226 | 253 | 182 | 183 |
| Polvere et al | 2023 | cohort study | Europe | BNT162b2 or mRNA1273 | 2 | after 2 months | 43 | 51 | 69 | 75 |
| Park et al | 2022 | cohort study | Asia | mRNA vaccine or viral vector vaccine | 2 | median 94 days in PLWH  median 162 days in HC | 14 | 14 | 218 | 224 |
| Park et al | 2022 | cohort study | Asia | mRNA vaccine or viral vector vaccine | 3 | at a median of 63 days | 29 | 29 | 114 | 114 |
| Hensley et al | 2022 | cohort study | Europe | mRNA vaccine | 2 | after 4 weeks | 971 | 984 | 341 | 341 |
| Hensley et al | 2022 | cohort study | Europe | Viral vector vaccine | 2 | after 4 weeks | 150 | 170 | 81 | 99 |
| Nault et al | 2022 | cohort study | North America | BNT162b2 or mRNA1273 | 1 | after 3-4 weeks | 100 | 106 | 19 | 20 |
| Sisteré-Oró et al | 2022 | cohort study | Europe | BNT162b2 | 2 | after 3 weeks | 5 | 10 | 10 | 10 |
| Oyaert et al | 2022 | cohort study | Europe | BNT162b2 | 2 | after 10-14 days | 23 | 23 | 52 | 52 |
| Oyaert et al | 2022 | cohort study | Europe | BNT162b2 | 1 | after 21-28 days | 23 | 27 | 54 | 54 |
| Brumme et al | 2022 | cohort study | North America | mRNA vaccine or viral vector vaccine | 1 | after 1 month | 89 | 90 | 132 | 134 |
| Brumme et al | 2022 | cohort study | North America | mRNA vaccine or viral vector vaccine | 2 | after 1 month | 91 | 91 | 136 | 136 |
| Frater et al | 2021 | non-randomized controlled trial | Europe | ChAdOx1 | 2 | after 14 days | 50 | 50 | 46 | 46 |

^*^the time between the last dose and antibody testing

**Supplementary Table S2:** Baseline Characteristics of HIV-negative individuals and PLWH in the included studies.

| Author | Age | | Gender  (percentage) male | | Median CD4+ T-cell count  (cells/µL) | Nadir CD4+ count  (cells/µL) | Percentage on ART therapy | Percentage with suppressed viral load |
| --- | --- | --- | --- | --- | --- | --- | --- | --- |
|  | PLWH | HC | PLWH | HC | PLWH | PLWH | PLWH | PLWH |
| Zou et al | median (IQR):  36 (31-42) | median (IQR):  31 (27-39) | 40/46 (87%) | 19/38 (50%) | 523 |  | 46/46  (100%) | 41/46  (89%) |
| Spinelli et al | median (IQR):  59 (50-66) | median (IQR):  59 (52-66) | 87/100 (87%) | 87/100 (87%) | 511 |  |  |  |
| Antinori et al | PCDR:  median (IQR):  57 (52-60)  ICDR:  median (IQR):  54 (46-59)  HCDR:  median (IQR):  54 (46-59) | median (IQR):  42 (32-53) | 139/166 (83.7%) | 121/169 (71.6%) | PCDR:  140  ICDR:  335  HCDR:  727 | PCDR:  median (IQR): 49 (23-122)  ICDR:  median (IQR): 63 (29-150)  HCDR:  median (IQR): 174 (68-280) | 166/166  (100%) | 152/166  (91.6%) |
| Han et al | median (IQR):  34 (26-42) | median (IQR):  37 (33-50) | 45/47 (95.7%) | 18/18 (100%) |  |  | 47/47  (100%) |  |
| Cai et al | mean (sd):  32.55 (8.69) | mean (sd):  29.84 (8.51) | 140/143 (97.9%) | 48/50 (96.0%) |  |  | 116/143  (81.1%) |  |
| Levy et al | mean (sd):  49.8 (11.6) | mean (sd):  55.8 (14.3) | 131/143 (91.6%) | 66/261 (25.3%) |  | mean (range):  345 (2-900) | 143/143  (100%) | 136/143  (95%) |
| Netto et al | median (IQR):  54 (45-60) | median (IQR):  48 (37-58) | 130/215 (60%) | 109/296 (37%) | 655 |  | 214/215  (99.5%) | 191/215  (89%) |
| Loubet et al | median (IQR):  55.3 (49.7-61.0) |  | 668/897 (74.5%) |  |  |  |  | 431/544  (79.2%) |
| Ao et al | median (range):  55 (23-81) | median (range):  54 (21-83) | 89/139 (64.0%) | 72/120 (60.0%) |  |  | 139/139  (100%) | 109/139  (78.4%) |
| Zeng et al | median (IQR):  32 (28-39) |  | 119/132 (90.2%) |  | 505 |  | 126/132  (95.5%) |  |
| Haidar et al | mean (sd):  57.4 (10.0) | mean (sd):  44.2 (13.3) | 84/94 (89.4%) | 43/172 (25.0%) |  |  | 94/94  (100%) | 92/94  (97.9%) |
| Heftdal et al | median (IQR):  56 (49-64) | median (IQR):  56 (49-63) | 242/269 (90.0%) | 73/538 (13.6%) | 640 | median (IQR):  246 (150-375) | 268/269  (99.6%) | 260/269  (96.7%) |
| Bergman et al |  |  | 54/90 (60%) | 39/90 (43%) |  |  |  |  |
| Lv et al | median (IQR):  44.00 (39.00-48.75) | median (IQR):  37.00 (26.25-47.25) | 12/24 (50.0%) | 15/24 (62.5%) |  |  |  |  |
| Feng et al | mean (sd):  42.74 (10.17) | mean (sd):  37.79 (8.80) | 29/42 (69.0%) | 16/28 (57.1%) |  |  | 42/42  (100%) | 30/42  (71.4%) |
| Huang et al | median (IQR):  34 (28-38) | median (IQR):  34 (29-47) | 128/129 (99.2%) | 40/53 (75.5%) | 630.5 |  | 126/129  (97.7%) | 75/129  (58.1%) |
| Rahav et al | median (IQR):  49 (42-57) |  | 137/156 (87.8%) |  |  |  |  | 95% |
| Cheng et al | mean (sd):  38.6 (13.1) | mean (sd):  42.8 (14.9) | 54/57 (94.7%) | 310/326 (95.1%) |  |  |  |  |
| Alessio et al | mean (sd):  48 (13.2) | mean (sd):  42.1 (12.1) | 85.1% | 80.5% |  |  | 84/84  (100%) | 86.9% |
| Madhi et al | median (IQR):  37 (32-45) | median (IQR):  34 (23-41) | 16/52 (31%) | 17/29 (59%) | 742 |  | 36/52  (69%) | 9/14  (64%) |
| Tortellini et al | median (IQR):  61 (48-68) | median (IQR):  30 (30-53) | 26/37 (70.3%) | 13/18 (72.2%) | 547 | median (IQR):  90 (22-281) | 37/37  (100%) |  |
| Costiniuk et al | median (IQR):  54.4 (42.3-62.8) | median (IQR):  42.0 (34.0-54.0) | 227/294 (77.2%) | 70/267 (26.2%) | 650 | median (IQR):  256 (120-444) | 287/294  (97.6%) |  |
| Wong et al | median (IQR):  45 (35-53) | median (IQR):  37 (33-45) | 535/593 (91%) | 42/84 (50%) | CoronaVac: 566  Comirnaty: 565 | median (IQR):  CoronaVac: 181 (44-338)  Comirnaty: 262 (125-386) |  | CoronaVac:  144/149 (97%)  Comirnaty:  310/327 (95%) |
| Vergori et al | HCDR:  median (IQR):  52 (47-58)  ICDR:  median (IQR):  55 (47-60)  PCDR:  median (IQR):  57 (48-63) |  | HCDR:  64/76 (84.2%)  ICDR:  79/96 (82.3%)  PCDR:  34/44 (77.3%) |  |  | HCDR: median (IQR):  83 (26-168)  ICDR: median (IQR):  41 (16-92)  PCDR: median (IQR):  40 (15-76) | HCDR:  76/76 (100%)  ICDR:  96/96 (100%)  PCDR:  44/44 (100%) | HCDR:  65/68 (95.6%)  ICDR:  80/91 (87.9%)  PCDR:  23/41 (56.1%) |
| Zhan et al |  |  | 294/318 (92.5%) |  |  |  | 318/318  (100%) |  |
| Polvere et al | median (IQR):  52 (46-58) | median (IQR):  52 (45-60) | 64/84 (76.2%) | 22/79 (27.8%) | 639 | median (IQR):  154 (34-302) | 84/84  (100%) | 76/84  (90.5%) |
| Park et al | median (IQR):  44 (34-56) | median (IQR):  35 (26-45) | 28/29 (96.6%) | 38/114 (33.3%) | 670 |  |  | 25/29  (86.2%) |
| Hensley et al | median (IQR):  53 (44-60) | median (IQR):  43 (33-53) | 987/1154 (85.5%) | 126/440 (28.6%) | 710 |  | 1142/1154  (99.0%) | 1127/1154  (97.7%) |
| Nault et al | mean (range):  43 (21-65) | mean (range):  47 (21-59) | 90/106 (84.9%) | 7/20 (35.0%) |  |  |  | 102/106  (96.2%) |
| Sisteré-Oró et  al | median: 50.5 |  | 7/10 (70%) |  | 169 | median: 63.5 | 10/10  (100%) | 8/10  (80%) |
| Oyaert et al | median (IQR):  47 (30-66) |  | 19/27 (70.4%) |  | 254 |  |  |  |
| Brumme et al | median (IQR):  54 (40-61) | median (IQR):  47 (35-70) | 88/100 (88%) | 50/152 (33%) | 710 | median (IQR):  280 (120-490) | 100/100  (100%) | 95/100  (95%) |
| Frater et al | median (IQR):  42.5 (37.2-49.8) | median (IQR):  38.5 (29.2-45.0) | 54/54 (100%) | 26/50 (52%) | 694 |  | 54/54  (100%) | 54/54  (100%) |

Abbreviations: PLWH: people living with HIV; PCDR: poor CD4 recovery ICDR: intermediate CD4 recovery HCDR: high CD4 recovery

**Supplementary Table S3:** Characteristics and basic information of the studies included in the systematic review and meta-analysis for vaccine safety.

| Author | Publication  year | Study design | Region | Risk of bias | Vaccine type | Vaccine  dose | Type of  adverse  events | PLWH with adverse events | PLWH | HC with adverse events | HC |
| --- | --- | --- | --- | --- | --- | --- | --- | --- | --- | --- | --- |
| Zou et al | 2022 | cohort study | Asia | Moderate | Sinopharm or WIBP-CorV | 1 | Total | 14 | 46 | 12 | 38 |
| Zou et al | 2022 | cohort study | Asia | Moderate | Sinopharm or WIBP-CorV | 1 | Local | 14 | 46 | 11 | 38 |
| Zou et al | 2022 | cohort study | Asia | Moderate | Sinopharm or WIBP-CorV | 1 | Systemic | 5 | 46 | 2 | 38 |
| Zou et al | 2022 | cohort study | Asia | Moderate | Sinopharm or WIBP-CorV | 2 | Total | 5 | 46 | 9 | 38 |
| Zou et al | 2022 | cohort study | Asia | Moderate | Sinopharm or WIBP-CorV | 2 | Local | 4 | 46 | 6 | 38 |
| Zou et al | 2022 | cohort study | Asia | Moderate | Sinopharm or WIBP-CorV | 2 | Systemic | 1 | 46 | 3 | 38 |
| Netto et al | 2022 | cohort study | South America | Low | CoronaVac | 1 | Total | 60 | 169 | 122 | 296 |
| Netto et al | 2022 | cohort study | South America | Low | CoronaVac | 1 | Local | 21 | 169 | 61 | 296 |
| Netto et al | 2022 | cohort study | South America | Low | CoronaVac | 1 | Systemic | 55 | 169 | 97 | 296 |
| Netto et al | 2022 | cohort study | South America | Low | CoronaVac | 2 | Total | 55 | 189 | 91 | 265 |
| Netto et al | 2022 | cohort study | South America | Low | CoronaVac | 2 | Local | 30 | 189 | 45 | 265 |
| Netto et al | 2022 | cohort study | South America | Low | CoronaVac | 2 | Systemic | 48 | 189 | 78 | 265 |
| Madhi et al | 2022 | RCT | Africa | Low | NVX-CoV2373 | 1 | Local | 20 | 79 | 427 | 1397 |
| Madhi et al | 2022 | RCT | Africa | Low | NVX-CoV2373 | 1 | Systemic | 20 | 79 | 401 | 1397 |
| Madhi et al | 2022 | RCT | Africa | Low | NVX-CoV2373 | 2 | Local | 25 | 78 | 393 | 1351 |
| Madhi et al | 2022 | RCT | Africa | Low | NVX-CoV2373 | 2 | Systemic | 12 | 78 | 336 | 1351 |
| Ao et al | 2022 | cohort study | Asia | Low | BBIBP-CorV or CoronaVac | 2 | Total | 18 | 139 | 16 | 120 |
| Rahav et al | 2021 | cohort study | Asia | Low | BNT162b2 | 1 | Local | 58 | 143 | 199 | 272 |
| Rahav et al | 2021 | cohort study | Asia | Low | BNT162b2 | 1 | Systemic | 28 | 143 | 57 | 272 |
| Rahav et al | 2021 | cohort study | Asia | Low | BNT162b2 | 2 | Local | 37 | 143 | 223 | 272 |
| Rahav et al | 2021 | cohort study | Asia | Low | BNT162b2 | 2 | Systemic | 68 | 143 | 150 | 272 |
| Frater et al | 2022 | non-randomized controlled trial | North America | Low | AZD1222 | 1 | Local | 40 | 53 | 44 | 50 |
| Frater et al | 2022 | non-randomized controlled trial | North America | Low | AZD1222 | 1 | Systemic | 40 | 53 | 43 | 50 |
| Frater et al | 2022 | non-randomized controlled trial | North America | Low | AZD1222 | 2 | Local | 24 | 51 | 37 | 49 |
| Frater et al | 2022 | non-randomized controlled trial | North America | Low | AZD1222 | 2 | Systemic | 22 | 51 | 32 | 49 |

**Supplementary Table S4:** Risk of bias of all included randomized controlled trials (RCT) using the RoB 2 scale.

| Author | Bias arising from the  randomization process | Bias due to deviations from  intended interventions | Bias due to missing  outcome data | Bias in measurement  of the outcome | Bias in selection of  the reported result | Overall bias |
| --- | --- | --- | --- | --- | --- | --- |
| Madhi et al | L | L | L | L | L | L |

L: Low risk of bias

**Supplementary Table S5:** Risk of bias of all included non-randomized controlled trials using the ROBINS-I scale.

| Author | Bias due to  confounding | Bias in selection  of participants  into the study | Bias in  classification of  interventions | Bias due to  deviations from  intended  interventions | Bias due to  missing data | Bias in  measurement of  outcomes | Bias in  selection of  the reported  result | Overall bias |
| --- | --- | --- | --- | --- | --- | --- | --- | --- |
| Bergman et al | L | L | L | L | L | L | L | L |
| Feng et al | L | L | L | L | L | L | L | L |
| Frater et al | L | L | L | L | L | L | L | L |
| Lv et al | L | L | L | L | L | L | L | L |

L: Low risk of bias

**Supplementary Table S6:** Risk of bias of all included cohort studies using the Newcastle-Ottawa quality assessment scale.

| Author | Selection | | | | Comparability | Outcome | | | Total  score | Overall bias* |
| --- | --- | --- | --- | --- | --- | --- | --- | --- | --- | --- |
|  | Representativeness of the Exposed Cohort | Selection of the  Non-Exposed  Cohort | Ascertainment of  Exposure | Demonstration That  Outcome of Interest  Was Not Present at Start of Study |  | Assessment  of Outcome | Was Follow-Up  Long Enough for  Outcomes to  Occur | Adequacy of  Follow Up of  Cohorts |  |  |
| Zou et al | 1 | 0 | 1 | 0 | 2 | 1 | 0 | 0 | 5 | M |
| Antinori et al | 1 | 0 | 1 | 1 | 2 | 1 | 1 | 1 | 8 | L |
| Han et al | 0 | 1 | 0 | 1 | 2 | 1 | 0 | 1 | 6 | M |
| Levy et al | 1 | 0 | 1 | 1 | 2 | 1 | 0 | 1 | 7 | L |
| Netto et al | 0 | 0 | 1 | 1 | 2 | 1 | 1 | 1 | 7 | L |
| Loubet et al | 1 | 1 | 0 | 1 | 2 | 1 | 0 | 1 | 7 | L |
| Ao et al | 1 | 0 | 1 | 1 | 2 | 1 | 1 | 0 | 7 | L |
| Zeng et al | 1 | 0 | 1 | 0 | 2 | 1 | 0 | 1 | 6 | M |
| Haidar et al | 1 | 1 | 0 | 1 | 2 | 1 | 1 | 1 | 8 | L |
| Heftdal et al | 1 | 1 | 1 | 0 | 1 | 1 | 1 | 1 | 7 | L |
| Rahav et al | 1 | 0 | 1 | 1 | 1 | 1 | 1 | 1 | 7 | L |
| Cheng et al | 1 | 0 | 0 | 1 | 1 | 1 | 0 | 1 | 5 | M |
| Tortellini et al | 1 | 0 | 1 | 1 | 1 | 1 | 1 | 0 | 6 | M |
| Costiniuk et al | 0 | 0 | 1 | 1 | 1 | 1 | 1 | 0 | 5 | M |
| Wong et al | 0 | 1 | 0 | 1 | 1 | 1 | 0 | 1 | 5 | M |
| Vergori et al | 1 | 1 | 1 | 1 | 2 | 1 | 0 | 1 | 8 | L |
| Polvere et al | 1 | 0 | 1 | 1 | 1 | 1 | 1 | 0 | 6 | M |
| Park et al | 1 | 0 | 0 | 1 | 2 | 1 | 0 | 0 | 5 | M |
| Hensley et al | 1 | 0 | 1 | 1 | 2 | 1 | 0 | 1 | 7 | L |
| Nault et al | 0 | 0 | 1 | 0 | 1 | 1 | 1 | 1 | 5 | M |
| Sisteré-Oró et al | 1 | 1 | 1 | 1 | 1 | 1 | 1 | 0 | 7 | L |
| Oyaert et al | 0 | 1 | 1 | 1 | 1 | 1 | 0 | 1 | 6 | M |
| Brumme et al | 0 | 0 | 1 | 0 | 2 | 0 | 1 | 0 | 4 | H |

*L: Low risk (total score ≥ 7), M: Moderate risk (total score 5-6), and H: High risk (total score ≤ 4).
